# Targeted Optical Imaging of the Glucagonlike Peptide 1 Receptor Using Exendin-4-IRDye 800CW

**DOI:** 10.2967/jnumed.119.234542

**Published:** 2020-07

**Authors:** Marti Boss, Desiree Bos, Cathelijne Frielink, Gerwin Sandker, Selen Ekim, Camille Marciniak, Francois Pattou, Go van Dam, Sanne van Lith, Maarten Brom, Martin Gotthardt, Mijke Buitinga

**Affiliations:** 1 Department of Radiology and Nuclear Medicine, Radboud University Medical Center, Nijmegen, The Netherlands; 2 Department of General and Endocrine Surgery, University Hospital 2 Lille, Lille, France; 3 Department of Surgery, University Medical Center Groningen, Groningen, The Netherlands; and; 4 Department of Clinical and Experimental Endocrinology, KU Leuven, Leuven, Belgium

**Keywords:** optical imaging, exendin, insulinoma, congenital hyperinsulinism, fluorescence

## Abstract

The treatment of choice for insulinomas and focal lesions in congenital hyperinsulinism (CHI) is surgery. However, intraoperative detection can be challenging. This challenge could be overcome with intraoperative fluorescence imaging, which provides real-time lesion detection with a high spatial resolution. Here, a novel method for targeted near-infrared (NIR) fluorescence imaging of glucagonlike peptide 1 receptor (GLP-1R)–positive lesions, using the GLP-1 agonist exendin-4 labeled with IRDye 800CW, was examined in vitro and in vivo. **Methods:** A competitive binding assay was performed using Chinese hamster lung (CHL) cells transfected with GLP-1R. Tracer biodistribution was determined in BALB/c nude mice bearing subcutaneous CHL-GLP-1R xenografts. In vivo NIR fluorescence imaging of CHL-GLP-1R xenografts was performed. Localization of the tracer in the pancreatic islets of BALB/c nude mice was examined using fluorescence microscopy. Laparoscopic imaging was performed to detect the fluorescent signal of the tracer in the pancreas of mini pigs. **Results:** Exendin-4-IRDye 800CW binds GLP-1R with a half-maximal inhibitory concentration of 3.96 nM. The tracer accumulates in CHL-GLP-1R xenografts. Subcutaneous CHL-GLP-1R xenografts were visualized using in vivo NIR fluorescence imaging. The tracer accumulates specifically in the pancreatic islets of mice, and a clear fluorescent signal was detected in the pancreas of mini pigs. **Conclusion:** These data provide the first in vivo evidence of the feasibility of targeted fluorescence imaging of GLP-1R–positive lesions. Intraoperative lesion delineation using exendin-4-IRDye 800CW could benefit open as well as laparoscopic surgical procedures for removal of insulinomas and focal lesions in CHI.

Although preoperative imaging is essential for tumor detection before surgical cancer treatment, translating this information into the operating room is often challenging. Intraoperative optical imaging can provide real-time detection of tumor lesions and thereby contribute to optimal surgical procedures (1).

Insulinomas, insulin-producing neuroendocrine tumors arising from stem cells or pancreatic β-cells, are the most common cause of endogenous adult hyperinsulinemic hypoglycemia (2). Persistent hypoglycemia also occurs in neonates and is in most cases caused by congenital hyperinsulinism (CHI). There are 2 subforms of this disease: focal CHI, caused by focal adenomatous islet cell hyperplasia, and diffuse CHI, resulting from diffuse involvement of pancreatic β-cells (3). Symptoms of insulinomas and CHI, caused by episodic hypoglycemia, are severe and include confusion, diplopia, and dizziness and, in cases of prolonged hypoglycemia, even seizure, loss of consciousness, or death (4).

Insulinomas and focal CHI can be completely cured by surgical removal of the lesion. However, these procedures are complicated by the usually small size of the lesions and their proximity to the pancreatic duct and major vessels (5). Precise localization of the lesion is of great importance and starts with sensitive preoperative detection. For insulinomas, this is performed using various imaging modalities: contrast-enhanced CT and MRI with sensitivities of around 70% and 90%, respectively (6); somatostatin receptor PET, for which sensitivities from 33% to 85% are reported (7,8); and the more invasive endoscopic ultrasound, with a sensitivity of 75% for detection of insulinomas (9). Currently, there is increasing evidence of the superior performance of the novel imaging method glucagonlike peptide 1 receptor (GLP-1R) SPECT/CT or PET/CT, using radiolabeled exendin-4, a stable analog of the hormone GLP-1, which specifically binds GLP-1R on pancreatic β-cells with high affinity (10). With this technique, insulinomas are detected with a sensitivity of up to 97.7% (7,11,12). Focal CHI is preoperatively localized using ^18^F-DOPA PET/CT with a sensitivity of 85% (13). GLP-1R PET is also being investigated as a potentially more sensitive imaging technique for focal CHI (NCT03768518).

However, even after preoperative visualization of the lesion, intraoperative detection can be challenging, especially in patients with multiple insulinomas, for which very small lesions (<1 cm) are even more common. Intraoperative ultrasound is routinely used for intraoperative localization of insulinomas. In combination with palpation, success rates ranging from 91% to 100% have been reported (9,14,15). However, a laparoscopic procedure, which is preferred when enucleation of the lesion is possible, excludes palpation. Radioguided detection of insulinomas has been proven successful in only a limited number of patients to date (16). Another interesting option for intraoperative detection of GLP-1R–positive lesions is fluorescence imaging, which has a better spatial resolution and could be used for precise delineation of the lesion and fluorescence-guided surgery (1,17).

Currently, there is much attention on the development of tracers for intraoperative fluorescence imaging, mostly using near-infrared (NIR) fluorophores, which have the benefit of a high penetration depth (5–10 mm) through tissue (18). Coupling of a fluorophore to a tumor-targeting moiety is crucial to ensure specific and efficient delivery of the fluorophore to the lesion of interest.

For targeting of insulinomas and focal CHI, the peptide exendin-4 is an attractive targeting agent for optical imaging.

We have developed exendin-4 coupled to the NIR fluorophore IRDye (Li-COR Biosciences) 800CW as a specific tracer for fluorescence imaging of insulinomas and focal CHI. We assessed the potential of targeting GLP-1R–positive cells in vitro and in vivo and the feasibility of performing in vivo fluorescence imaging with this compound.

## MATERIALS AND METHODS

### Reagents

Exendin-4-IRDye 800CW was supplied by piCHEM. IRDye 800CW NHS ester was obtained from Li-COR Biosciences. The N-ε-amino group of lysine at position 40 was site-specifically modified during solid-phase peptide synthesis with a mercaptopropionic acid, releasing an unprotected exendin-4 with a free thiol function after triisopropylsilane cleavage. IRDye 800CW was modified with a maleimide, and coupling to exendin-4 was performed using a thiol-reactive crosslinking approach. The purity was more than 90%. Stock solutions of exendin-4-IRDye 800CW were prepared in phosphate-buffered saline (PBS).

### Cell Culture

Chinese hamster lung (CHL) cells stably transfected with human GLP-1R (19) were cultured (at 37°C and 5% CO_2_) in Dulbecco modified Eagle medium (Thermo Fisher Scientific) with 4.5 g/L d-glucose and GlutaMAX (Life Technologies Corp.), supplemented with 10% fetal calf serum (Life Technologies), 100 IU/mL penicillin G, 10 mg/mL streptomycin, 1 mM sodium pyruvate, 0.1 mM nonessential amino acids, and 0.5 mg/mL G418 geneticin.

### Competitive Binding Assay

The half-maximal inhibitory concentration (IC_50_) of exendin-4-IRDye 800CW, and of unlabeled exendin as a reference, was determined using CHL-GLP-1R cells in a competitive binding assay as described previously (10,20). Cells were grown overnight in 6-well plates (∼10^6^ cells/well). Exendin-3-diethylenetriaminpentaacetic acid (DTPA), labeled with ^111^In as described earlier (21), was used as a tracer. The cells were washed twice with PBS and incubated for 4 h on ice with 50,000-cpm ^111^In-DTPA-exendin-3 in the presence of increasing concentrations of exendin-4-IRDye 800CW or exendin-4 (0.1–300 nM). After incubation, the cells were washed with PBS, solubilized with 2 mL of sodium hydroxide (NaOH), and collected, and the cell-associated activity was measured in a γ-counter (Wizard 2480; PerkinElmer).

### Binding Assay

The receptor specificity of the binding of the exendin-4-IRDye 800CW to CHL-GLP-1R cells was determined in a fluorescent binding assay. CHL-GLP-1R cells were grown overnight in 24-well plates to approximately 95% confluency. Cells were washed twice with binding buffer (Dulbecco modified Eagle medium supplemented with 0.5% bovine serum albumin) and incubated with 300 nM exendin-4-IRDye 800CW in triplicate with and without a ×50 excess of unlabeled exendin-4 (4 h at 37°C). After incubation, the cells were washed twice with PBS, lysed using 200 μL of sodium hydroxide per well, collected, and transferred to a black flat-bottom 96-well plate. Fluorescence was measured using a plate reader (Infinite Pro M200; Tecan Austria GmbH) (excitation, 750 nm; emission, 795 nm). Standard curves were created, and binding percentages to the cells were calculated using Excel (version 2007; Microsoft).

### Animal Tumor Model

Female BALB/c nude mice (Janvier) (age, 6–8 wk) were housed in individually ventilated cages (6 mice per cage) under nonsterile conditions with ad libitum access to chlorophyll-free animal chow and water. CHL-GLP-1R cells were injected subcutaneously on the right shoulder (5 × 10^6^ cells/mouse) in 200 μL of Dulbecco modified Eagle medium with 4.5 g/L d-glucose and GlutaMAX. All animal experiments were approved by the institutional Animal Welfare Committee of the Radboud University Medical Centre and were conducted in accordance with the guidelines of the Revised Dutch Act on Animal Experimentation.

### In Vivo Biodistribution

Female BALB/c nude mice bearing subcutaneous CHL-GLP-1R xenografts were injected intravenously with various concentrations of exendin-4-IRDye 800CW in 200 μL of PBS with 0.5% bovine serum albumin (*n* = 6 mice per group; 3, 10, 30, and 100 μg of exendin-4-IRDye 800CW). Control mice (*n* = 4) were injected with PBS with 0.5% bovine serum albumin only. After 4 h, the mice were sacrificed by CO_2_ asphyxiation, and blood and organs were removed and collected in MagNA Lyser tubes (F. Hoffmann- La Roche Ltd.), which were weighed before and after organ collection. The circulation time of 4 h was based on our previous experience with radiolabeled exendin tracers (10). Radioimmunoprecipitation assay lysis buffer (500 μL; 50 mM [hydroxymethyl]aminomethane-hydrochloride, pH 7.4, with 150 mM sodium chloride, 1 mM ethylenediaminetetraacetic acid, 1% Triton X-100 [Dow Chemical Co.], and 1% sodium dodecyl sulphate) was added to each tube. Organs were then homogenized using a MagNA Lyser with repeated cycles of 6,000 rpm for 25 s and cooling on ice for 1 min after each cycle. Organ homogenates of control mice were used to create standard curves for each organ. Organ homogenates (100 μL) and the standards were transferred in triplicate to a black flat-bottom 96-well plate. Fluorescence was measured using an Infinite Pro M200 (excitation, 750 nm; emission, 795 nm). Standard curves were created, and tracer uptake in the various organs was calculated using Excel. Tracer uptake in each organ type was corrected for the weight of the dissected organ. To determine the specificity of tumor uptake, an additional biodistribution experiment was performed with 2 groups of mice (*n* = 6 per group), which were injected with 3 μg of exendin-4-IRDye 800CW, with coinjection of 150 μg of unlabeled exendin-4 in 1 of the groups.

We validated the biodistribution of the fluorescent tracer using a dual-labeled version of the exendin tracer DTPA-exendin-4 (piCHEM), labeled with both ^111^In and Cy5.5, as previously described (21). We calculated the tracer uptake in the organs using fluorescence as well as radioactive measures and compared the resulting values with each other (Supplemental Fig. 1).

### In Vivo Fluorescence Imaging of GLP-1R–Positive Tumors

To show the feasibility of visualizing GLP-1R–positive tumors using in vivo fluorescence imaging, BALB/c nude mice bearing subcutaneous CHL-GLP-1R xenografts were injected intravenously with 3 μg of exendin-4-IRDye 800CW (*n* = 3 per group). One group of mice was coinjected with an excess (150 μg) of unlabeled exendin-4. After 4 h, fluorescence imaging was performed using an IVIS Lumina closed-cabinet fluorescence scanner (Caliper LifeSciences) (excitation, 745 nm; autofluorescence correction excitation, 640 nm; both measured with the indocyanine green filter). After resection of the tumor lesions, the mice were imaged again. Subsequently, the mice were dissected to remove the pancreas. Pancreata were fixed overnight in 4% formalin and embedded in paraffin for fluorescence microscopy and immunohistochemistry.

### Fluorescence Microscopy and Immunohistochemistry

Sections of 4 μm were cut at 2 levels, 100 μm apart. One section of each level was deparaffinated in xylene for 2 min, after which fluorescence imaging was performed using an Odyssey CLx flatbed fluorescence scanner (800-nm channel; recording time, 1–5 min; focus, 1.0 mm; Li-COR Biosciences). Subsequently, these sections were stained for insulin as previously described (22). Consecutive sections were used for fluorescence microscopy. After deparaffination in xylene (2 times for 5 min), the cell nuclei were stained with Hoechst (33258; Invitrogen). Sections were mounted under a glass cover in modified Kaiser glycerine. Fluorescence imaging was performed using an inverted microscope (DMI6000B; Leica Biosystems) equipped with an NIR light source ranging up to 900 nm (X-Cite 200DC; Excelas Excelitas Technologies), an NIR filter set (microscope 2 band-pass filters 850–890 m–2p and long-pass emission filter HQ800795LP; Chroma Technology Corp.), a monochrome DFC365 FX fluorescence camera (one·4-million-pixel charge-coupled device; Leica Biosystems GmbH), and LAS-X software (Leica Biosystems GmbH).

### Laparoscopic Imaging of Pancreatic Tracer Uptake in Mini Pigs

The possibility of visualizing tracer uptake in the pancreatic β-cells in vivo using a laparoscopic laser device was assessed in mini pigs at the Department of Experimental Research of the Lille 2 University Facilities. Surgical interventions were approved by the local ethics committee. Three healthy adult Göttingenlike mini pigs (*Sus scrofa*; L’Institut de l’Elevage) were anesthetized using 4% isoflurane (Aerrane) after receiving premedication (intramuscular injection of ketamine [Ketamine1000; Virbac], 10 mg/kg of body weight, and xylazine [Sédaxylan; CEVA Santé Animale], 2.5 mg/kg of body weight). The mini pigs were infused with exendin-4-IRDye 800CW, 1.3 μg/kg, in 20 mL of PBS over 30 min through an intravenous catheter in the external jugular vein. Four hours after tracer injection, laparoscopic surgery was performed to access the pancreas, on which fluorescence imaging of the head and tail was performed using a laser device emitting light at 800 nm and a fluorescence camera (SurgVision BV).

### Statistical Analysis

Statistical calculations were performed using Prism (GraphPad Software). IC_50_ values were calculated by fitting the data with nonlinear regression using least-squares fit with Prism.

## RESULTS

### Exendin-4-IRDye 800CW Specifically Binds GLP-1R with High Affinity

**FIGURE 1.**
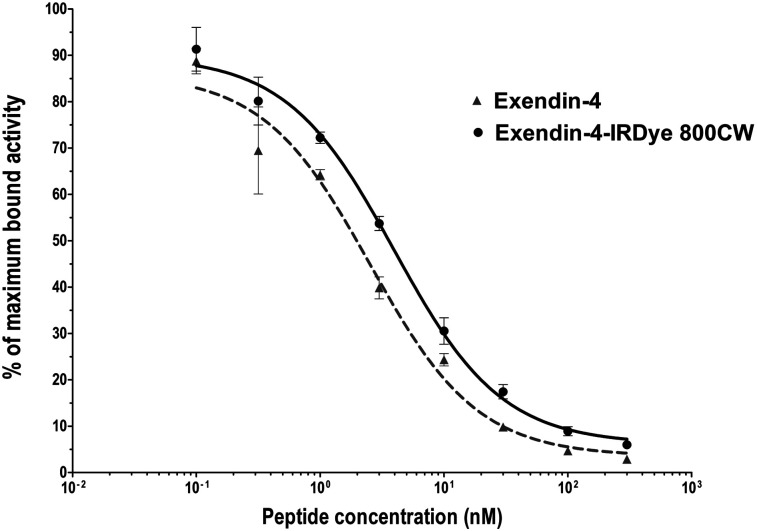
Competition binding assay (IC_50_) of unlabeled exendin-4 and exendin-4-IRDye 800CW on CHL-GLP-1R cells.

The IC_50_ values of unlabeled exendin-4 and exendin-4-IRDye 800CW were 2.5 nM (95% confidence interval, 1.32–4.90 nM) and 4.0 nM (95% confidence interval, 2.9–5.5 nM), respectively (Fig. 1). Although the binding affinity of the labeled peptide is significantly lower than that of the unlabeled peptide (*P* < 0.01), the binding affinity is in the same nanomolar range.

Addition of an excess of unlabeled exendin-4 decreased binding of exendin-4-IRDye 800CW to CHL-GLP-1R cells from 4.1% ± 0.4% to 0.3% ± 0.2% (*P* < 0.01) (Supplemental Fig. 2A).

### Exendin-4-IRDye 800CW Accumulates in CHL-GLP-1R Tumors

**FIGURE 2.**
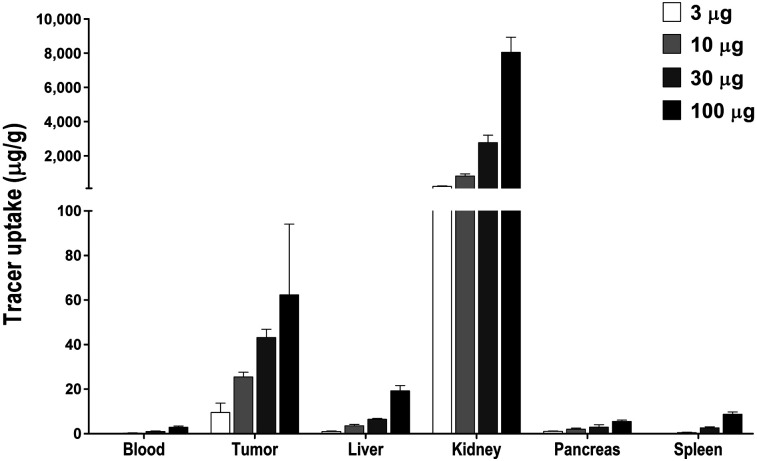
Biodistribution of exendin-IRDye 800CW in blood and various tissues of female BALB/c nude mice carrying subcutaneous CHL-GLP-1R tumors (*n* = 6/group) at 4 h after tracer injection.

Absolute tumor uptake of exendin-4-IRDye 800CW was 9.6 ± 4.2 μg of tracer/g of organ with an injected dose of 3 μg and increased dose-dependently to 25.5 ± 2.1 μg/g, 43.2 ± 3.6 μg/g, and 62.4 ± 31.8 μg/g with injected doses of 10, 30, and 100 μg, respectively. The highest uptake of exendin-4-IRDye 800CW was observed in the kidneys, because of renal clearance of the tracer (Fig. 2).

Coinjection of an excess of unlabeled exendin-4 decreased the tumor uptake of exendin-4-IRDye 800CW from 11.6 ± 1.6 %ID/g to 1.3 ± 0.4 %ID/g (*P* < 0.001) (Supplemental Fig. 2B).

### In Vivo Fluorescent Tumor Detection Is Feasible

**FIGURE 3.**
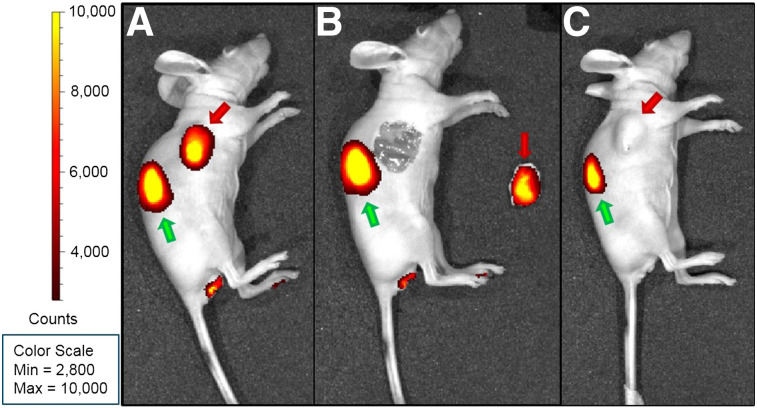
NIR fluorescence images of BALB/c nude mice bearing subcutaneous CHL-GLP1-R tumors. Tumors are indicated with red arrows and kidneys with green arrows. (A) Intact mouse. (B) Mouse after resection of tumor. (C) Mouse injected with exendin-4-IRDye 800CW and excess of unlabeled peptide.

Tumors were clearly visualized by fluorescence imaging (Fig. 3A; Supplemental Fig. 3). After resection of the tumors, no residual fluorescent signal was detected (Fig. 3B). Besides the signal in the tumors, a fluorescent signal was observed in the kidneys. In the mice receiving an excess of the unlabeled peptide, no fluorescent signal was seen in the tumors whereas the signal in the kidneys persisted, demonstrating the receptor specificity of the uptake of exendin-4-IRDye 800CW (Fig. 3C).

### Exendin-4-IRDye 800CW Accumulates Specifically in Murine Pancreatic Islets of Langerhans

**FIGURE 4.**
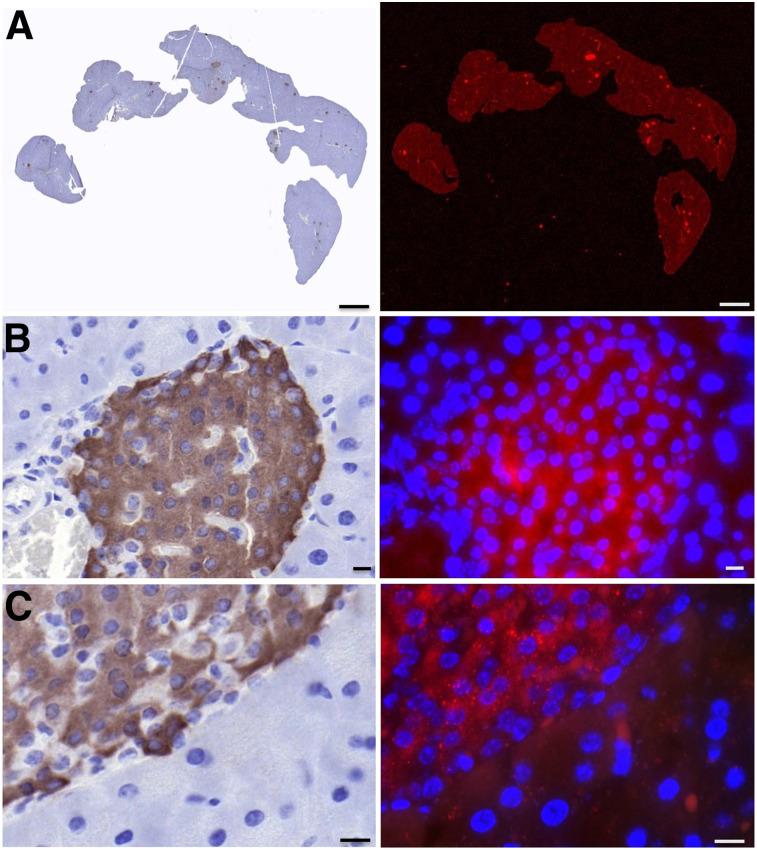
Immunohistochemistry flat-bed fluorescence (A) and fluorescence microscopy (B and C) images of pancreatic tissue of mouse injected with exendin-4-IRDye 800CW. Insulin staining is shown in brown (left), 800-nm fluorescent signal in red (right), and nuclei in blue (right). Microscopy images are at ×40 (B) and ×63 (C) magnification. Scale bars indicate 1,000 μm (A) or 10 μm (B and C).

Representative images of the islets of one of the mice are shown in Figure 4. The presence of pancreatic islets was clearly indicated by the positive insulin staining. A clear NIR fluorescent signal was observed at the site of the pancreatic islets. The signal appeared to be mostly intracellular, which corresponds with the fast internalization of exendin-4–based tracers. A much lower signal was observed in the exocrine pancreatic tissue. The higher fluorescent signal in the pancreatic islets points to receptor specificity of the uptake of exendin-4-IRDye 800CW.

### Pancreatic Uptake of Exendin-4-IRDye 800CW Is Detected by In Vivo Laparoscopic Imaging in Mini Pigs

**FIGURE 5.**
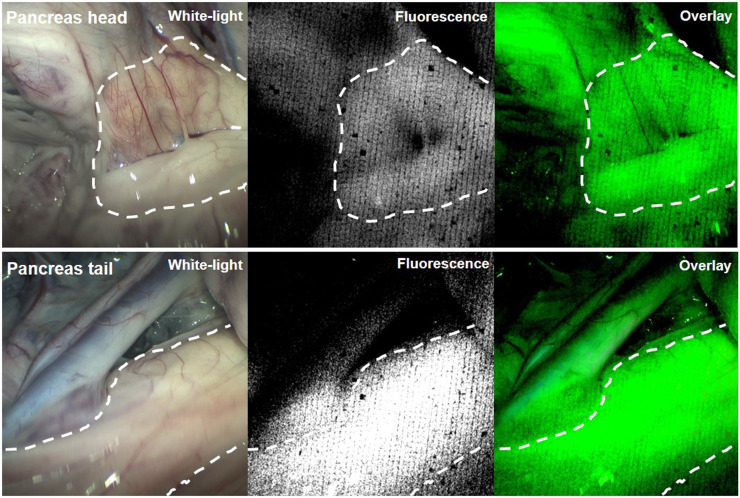
Laparoscopic images of pancreatic head and tail of healthy mini pig. On left are white-light images, in middle NIR fluorescent images, and on right layover images in which fluorescent signal is depicted in green.

Laparoscopic NIR fluorescence imaging of the pancreas in healthy mini pigs revealed a clear fluorescent signal in the pancreatic head and tail, with which it was possible to discriminate between pancreatic tissue and surrounding tissues (Fig. 5).

## DISCUSSION

Surgery to remove insulinomas or lesions in focal CHI is challenging and carries substantial risks of morbidity (4,5). Precise detection of the lesion is essential for an optimal surgical procedure. Real-time intraoperative detection with a high sensitivity and spatial resolution for precise lesion delineation can be provided by targeted fluorescence imaging. An agent for targeted NIR fluorescence imaging of the GLP-1R was developed and examined in vitro and in vivo in this study.

Exendin-4-IRDye 800CW was shown to have a high affinity for GLP-1R. Because of the high target affinity of the tracer, combined with fast clearance from nontarget tissues, a signal with a high target-to-background ratio could be achieved.

Exendin-4-IRDye 800CW was shown to accumulate dose-dependently in subcutaneous GLP-1R–positive tumors in mice. We demonstrated the reliability of the method used to assess the biodistribution of the fluorescent tracer by showing comparable results between fluorescent and radioactive quantification of uptake of the dual-labeled tracer ^111^In-DTPA-exendin-4-Cy5.5 in GLP-1R–positive tumors, pancreas, and kidneys of mice. We furthermore showed that with exendin-4-IRDye 800CW, subcutaneous GLP-1R–positive tumors could be visualized using NIR fluorescence imaging. Coinjection of an excess of unlabeled exendin-4 completely abolished the fluorescent signal in the tumors, demonstrating the receptor specificity of the tumor uptake. Furthermore, fluorescence microscopy showed uptake of the tracer specifically in the pancreatic islets of these mice.

The first report on intraoperative imaging of insulinomas involved a nontargeted approach based on the NIR dye methylene blue, which showed higher uptake in insulinomas than in normal pancreatic tissue at the proper dilution (23). A targeted approach, as described in this study, has the benefit of creating signals with higher contrast between the target and surrounding tissue and therefore more precise delineation of the lesion. Exendin-4 has already been shown effective for targeting of GLP-1R–positive tumors in preclinical and clinical studies using radiolabeled exendin (7,10,11). Reiner et al. have previously developed NIR imaging agents, based on exendin-4, for in vivo quantification of β-cell mass. These tracers were shown to bind GLP-1R with high affinity (IC_50_, 0.3–3 nM) and a fluorescent signal was observed in the pancreatic islets. However, uptake of these tracers in GLP-1R–positive tumors leading to a possible application for fluorescent-guided surgery was not assessed (24,25). Exendin-4 was used by Brand et al. to develop a dual-labeled tracer (^64^Cu-exendin-4-Cy5) for combined PET and fluorescence imaging of GLP-1R–positive tumors. Although the affinity of this tracer for GLP-1R (IC_50_, 50 nM) was lower than that of exendin-4-IRDye 800CW, specific accumulation in GLP-1R tumors was shown and GLP-1R–positive xenografts were visualized using in vivo PET imaging. However, although a fluorescent signal in the xenografts was detected using fluorescence microscopy, in vivo fluorescent imaging was not performed (26). We therefore here provide the first evidence, to our knowledge, of the feasibility of targeted in vivo fluorescence imaging of GLP-1R–positive lesions.

Using a laparoscopic NIR fluorescence imaging device, a fluorescent signal from exendin-4-IRDye 800CW in the pancreas of mini pigs could be detected. Since the exocrine pancreas in pigs is known to have a relatively high GLP-1R density resulting in a low endocrine-to-exocrine ratio of GLP-1R expression (27), the detected signal most probably does not originate only from the pancreatic islets. In mice, in which fluorescence microscopy showed specific uptake of the tracer in the pancreatic islets, the endocrine-to-exocrine ratio of GLP-1R expression is known to be much higher (27). However, even if the NIR fluorescent signal detected with the laparoscope originates from both the endocrine and the exocrine pancreas of mini pigs, the feasibility of using this laparoscopic procedure to detect NIR fluorescent signal clearly demonstrates the potential for clinical translation of this approach. Despite the background fluorescence signal in the pancreas resulting from uptake of the tracer in healthy pancreatic β-cells, visualization and delineation of insulinomas in humans using this approach is most likely possible, since insulinomas have a high GLP-1R density in almost 100% of cases (higher than healthy pancreatic islets) (28) and visualization with high tumor-to-background ratios has been achieved with radiolabeled exendin (7).

Contributing to the potential for clinical translation of this tracer is the use of the NIR fluorophore IRDye 800CW. This dye is widely used in the development of targeted fluorescence imaging approaches, and several studies already have shown the first successful clinical applications using this fluorophore. Detection of primary breast cancer lesions, as well as peritoneal metastases of colorectal cancer, was shown to be feasible using IRDye 800CW coupled to the monoclonal antibody bevacizumab (29,30). Also, IRDye 800CW coupled to the monoclonal antibody cetuximab was successfully used for detection of glioblastomas (31). Although the first successful clinical results with targeted NIR intraoperative imaging were obtained with monoclonal antibody–based approaches, various peptide-based NIR imaging approaches have been developed and demonstrated to be successful in preclinical settings for a wide range of cancer types. Also, several clinical trials are ongoing (32).

## CONCLUSION

We here show the feasibility of in vivo fluorescence imaging of GLP-1R–positive lesions using the novel tracer exendin-4-IRDye 800CW. Although applicable in open as well as laparoscopic procedures, this approach could be especially beneficial for laparoscopic procedures, in which surgeons currently rely solely on intraoperative ultrasound. In the future, fluorescence imaging using exendin-4-IRDye 800CW could benefit surgical removal of insulinomas and focal lesions in CHI by providing sensitive and specific real-time intraoperative optical lesion delineation.

## DISCLOSURE

This work is supported by BetaCure (FP7/2014-2018, grant agreement 602812). Martin Gotthardt is an inventor and holder of the patent “Invention Affecting GLP-1 and Exendin” (Philipps-Universität Marburg, June 17, 2009). No other potential conflict of interest relevant to this article was reported.

**KEY POINTS**

**QUESTION:** Is targeted fluorescence imaging a feasible technique to improve intraoperative detection of insulinomas and focal lesions in CHI?
**PERTINENT FINDINGS:** Exendin-4-IRDye 800CW binds GLP-1R with an IC_50_ of 3.96 nM. The tracer accumulates in CHL-GLP-1R xenografts. Subcutaneous CHL-GLP-1R xenografts were visualized using in vivo NIR fluorescence imaging, and no signal remained after fluorescence-guided resection. The tracer accumulates specifically in the pancreatic islets of mice, and a clear fluorescent signal was detected in the pancreas of mini pigs.
**IMPLICATIONS FOR PATIENT CARE:** Targeted optical imaging of GLP-1R–positive lesions could benefit surgical treatment of insulinomas and CHI by providing sensitive and specific real-time intraoperative optical lesion delineation.

## References

[1] de Boer E Harlaar NJ Taruttis A Optical innovations in surgery. Br J Surg. 2015;102:e56–e72. 25627136 10.1002/bjs.9713

[2] Kinova MK Diagnostics and treatment of insulinoma. Neoplasma. 2015;62:692–704. 26278152 10.4149/neo_2015_083

[3] Lord K Dzata E Snider KE Gallagher PR De Leon DD Clinical presentation and management of children with diffuse and focal hyperinsulinism: a review of 223 cases. J Clin Endocrinol Metab. 2013;98:E1786–E1789. 24057290 10.1210/jc.2013-2094 PMC3816257

[4] Senniappan S Shanti B James C Hussain K Hyperinsulinaemic hypoglycaemia: genetic mechanisms, diagnosis and management. J Inherit Metab Dis. 2012;35:589–601. 22231386 10.1007/s10545-011-9441-2

[5] Richards ML Gauger PG Thompson NW Kloos RG Giordano TJ Pitfalls in the surgical treatment of insulinoma. Surgery. 2002;132:1040–1049. 12490853 10.1067/msy.2002.128610

[6] Zhu L Xue H Sun Z Prospective comparison of biphasic contrast-enhanced CT, volume perfusion CT, and 3 Tesla MRI with diffusion-weighted imaging for insulinoma detection. J Magn Reson Imaging. 2017;46:1648–1655. 28419614 10.1002/jmri.25709

[7] Antwi K Fani M Heye T Comparison of glucagon-like peptide-1 receptor (GLP-1R) PET/CT, SPECT/CT and 3T MRI for the localisation of occult insulinomas: evaluation of diagnostic accuracy in a prospective crossover imaging study. Eur J Nucl Med Mol Imaging. 2018;45:2318–2327. 30054698 10.1007/s00259-018-4101-5

[8] Prasad V Sainz-Esteban A Arsenic R Role of ^68^Ga somatostatin receptor PET/CT in the detection of endogenous hyperinsulinaemic focus: an explorative study. Eur J Nucl Med Mol Imaging. 2016;43:1593–1600. 26923247 10.1007/s00259-016-3331-7

[9] Mehrabi A Fischer L Hafezi M A systematic review of localization, surgical treatment options, and outcome of insulinoma. Pancreas. 2014;43:675–686. 24921202 10.1097/MPA.0000000000000110

[10] Brom M Joosten L Oyen WJ Gotthardt M Boerman OC Radiolabelled GLP-1 analogues for in vivo targeting of insulinomas. Contrast Media Mol Imaging. 2012;7:160–166. 22434628 10.1002/cmmi.475 PMC4246000

[11] Christ E Wild D Ederer S Glucagon-like peptide-1 receptor imaging for the localisation of insulinomas: a prospective multicentre imaging study. Lancet Diabetes Endocrinol. 2013;1:115–122. 24622317 10.1016/S2213-8587(13)70049-4

[12] Luo Y Pan Q Yao S Glucagon-like peptide-1 receptor PET/CT with ^68^Ga-NOTA-exendin-4 for detecting localized insulinoma: a prospective cohort study. J Nucl Med. 2016;57:715–720. 26795291 10.2967/jnumed.115.167445 PMC5227553

[13] Laje P States LJ Zhuang H Accuracy of PET/CT scan in the diagnosis of the focal form of congenital hyperinsulinism. J Pediatr Surg. 2013;48:388–393. 23414871 10.1016/j.jpedsurg.2012.11.025 PMC3597386

[14] Fendrich V Bartsch DK Langer P Zielke A Rothmund M Diagnosis and surgical treatment of insulinoma: experiences in 40 cases [in German]. Dtsch Med Wochenschr. 2004;129:941–946. 15083396 10.1055/s-2004-823060

[15] Kisker O Bastian D Frank M Rothmund M Diagnostic localization of insulinoma: experiences with 25 patients with solitary tumors [in German]. Med Klin. 1996;91:349–354. 8767307

[16] Christ E Wild D Forrer F Glucagon-like peptide-1 receptor imaging for localization of insulinomas. J Clin Endocrinol Metab. 2009;94:4398–4405. 19820010 10.1210/jc.2009-1082

[17] DeLong JC Hoffman RM Bouvet M Current status and future perspectives of fluorescence-guided surgery for cancer. Expert Rev Anticancer Ther. 2016;16:71–81. 26567611 10.1586/14737140.2016.1121109 PMC5572645

[18] Ash C Dubec M Donne K Bashford T Effect of wavelength and beam width on penetration in light-tissue interaction using computational methods. Lasers Med Sci. 2017;32:1909–1918. 28900751 10.1007/s10103-017-2317-4 PMC5653719

[19] van Eyll B Lankat-Buttgereit B Bode HP Goke R Goke B Signal transduction of the GLP-1-receptor cloned from a human insulinoma. FEBS Lett. 1994;348:7–13. 7517895 10.1016/0014-5793(94)00553-2

[20] Jodal A Lankat-Buttgereit B Brom M Schibli R Behe M A comparison of three ^67/68^Ga-labelled exendin-4 derivatives for beta-cell imaging on the GLP-1 receptor: the influence of the conjugation site of NODAGA as chelator. EJNMMI Res. 2014;4:31. 25006548 10.1186/s13550-014-0031-9 PMC4078388

[21] Brom M Oyen WJ Joosten L Gotthardt M Boerman OC ^68^Ga-labelled exendin-3, a new agent for the detection of insulinomas with PET. Eur J Nucl Med Mol Imaging. 2010;37:1345–1355. 20111963 10.1007/s00259-009-1363-y PMC2886138

[22] Brom M Woliner-van der Weg W Joosten L Non-invasive quantification of the beta cell mass by SPECT with ^111^In-labelled exendin. Diabetologia. 2014;57:950–959. 24488022 10.1007/s00125-014-3166-3

[23] Winer JH Choi HS Gibbs-Strauss SL Ashitate Y Colson YL Frangioni JV Intraoperative localization of insulinoma and normal pancreas using invisible near-infrared fluorescent light. Ann Surg Oncol. 2010;17:1094–1100. 20033320 10.1245/s10434-009-0868-8 PMC2841719

[24] Reiner T Kohler RH Liew CW Near-infrared fluorescent probe for imaging of pancreatic beta cells. Bioconjug Chem. 2010;21:1362–1368. 20583828 10.1021/bc100184w PMC2912453

[25] Reiner T Thurber G Gaglia J Accurate measurement of pancreatic islet beta-cell mass using a second-generation fluorescent exendin-4 analog. Proc Natl Acad Sci USA. 2011;108:12815–12820. 21768367 10.1073/pnas.1109859108 PMC3150928

[26] Brand C Abdel-Atti D Zhang Y In vivo imaging of GLP-1R with a targeted bimodal PET/fluorescence imaging agent. Bioconjug Chem. 2014;25:1323–1330. 24856928 10.1021/bc500178d PMC4215873

[27] Eriksson O Rosenstrom U Selvaraju RK Eriksson B Velikyan I Species differences in pancreatic binding of DO3A-VS-Cys^40^-Exendin4. Acta Diabetol. 2017;54:1039–1045. 28891030 10.1007/s00592-017-1046-2 PMC5643362

[28] Reubi JC Waser B Concomitant expression of several peptide receptors in neuroendocrine tumours: molecular basis for in vivo multireceptor tumour targeting. Eur J Nucl Med Mol Imaging. 2003;30:781–793. 12707737 10.1007/s00259-003-1184-3

[29] Harlaar NJ Koller M de Jongh SJ Molecular fluorescence-guided surgery of peritoneal carcinomatosis of colorectal origin: a single-centre feasibility study. Lancet Gastroenterol Hepatol. 2016;1:283–290. 28404198 10.1016/S2468-1253(16)30082-6

[30] Lamberts LE Koch M de Jong JS Tumor-specific uptake of fluorescent bevacizumab-IRDye800CW microdosing in patients with primary breast cancer: a phase I feasibility study. Clin Cancer Res. 2017;23:2730–2741. 28119364 10.1158/1078-0432.CCR-16-0437

[31] Miller SE Tummers WS Teraphongphom N First-in-human intraoperative near-infrared fluorescence imaging of glioblastoma using cetuximab-IRDye800. J Neurooncol. 2018;139:135–143. 29623552 10.1007/s11060-018-2854-0 PMC6031450

[32] Joshi BP Wang TD Targeted optical imaging agents in cancer: focus on clinical applications. Contrast Media Mol Imaging. 2018;2018:2015237. 30224903 10.1155/2018/2015237 PMC6129851

