# Supplementary material for: Targeted Optical Imaging of the Glucagonlike Peptide 1 Receptor Using Exendin-4-IRDye 800CW
Source: J Nucl Med. 2020 Jul;61(7):1066–71. doi: 10.2967/jnumed.119.234542 (PMC7383075; doi:10.2967/jnumed.119.234542)
Supplement: Supplementary file 1 [file jnm234542SupplementalData.pdf]

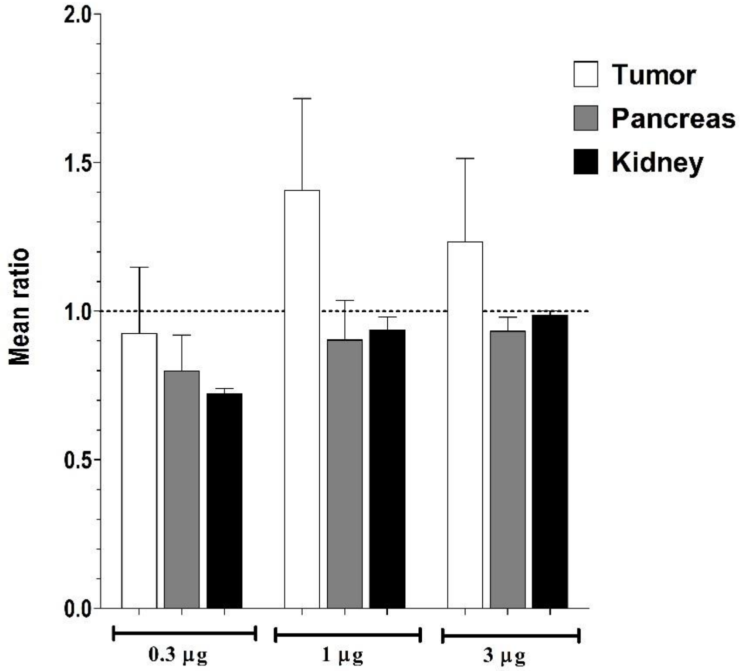

Supplemental Figure 1: Biodistribution of ^{111}In-DTPA-exendin-4-Cy5.5 (0.3 µg, 1 µg and 3 µg, N=6 mice per group) in tumor, pancreas and kidney. Uptake in organs were determined with radiochemical and fluorescent analysis. Given are the ratios of uptake values determined by the different methods showing a good correlation between the methods.

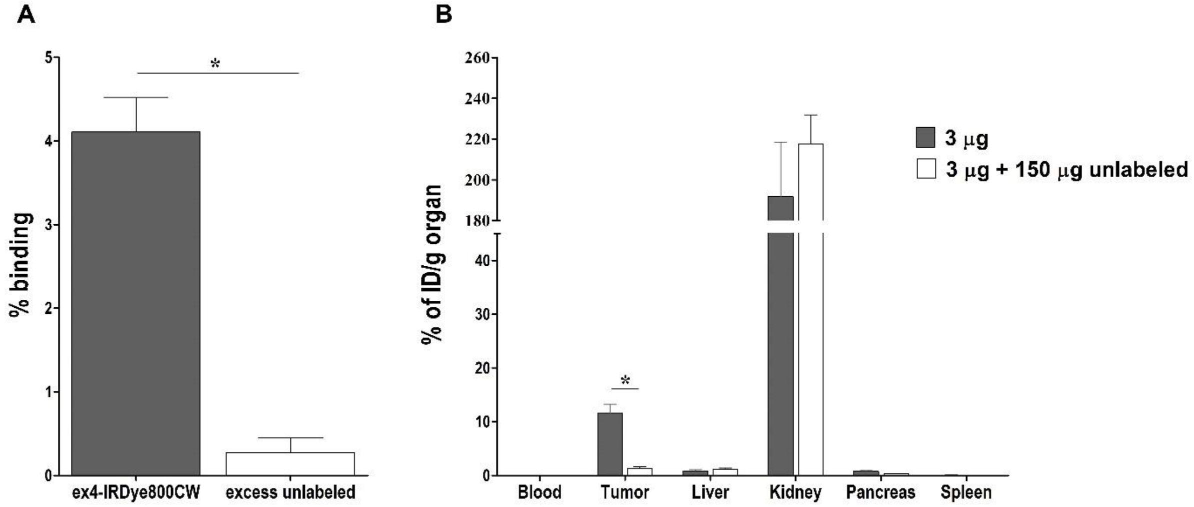

Supplemental Figure 2: Binding of exendin-4-IRDye800CW (300nM) to CHL-GLP-1R cells with and without an excess of unlabeled exendin-4 (N=3) (A). Biodistribution of exendin-IRDye800CW with and without an excess of unlabeled exendin-4 in blood and various tissues of female BALB/c nude mice carrying subcutaneous CHL-GLP-1R tumors (N=6/group) at 4 hours after tracer injection (B).

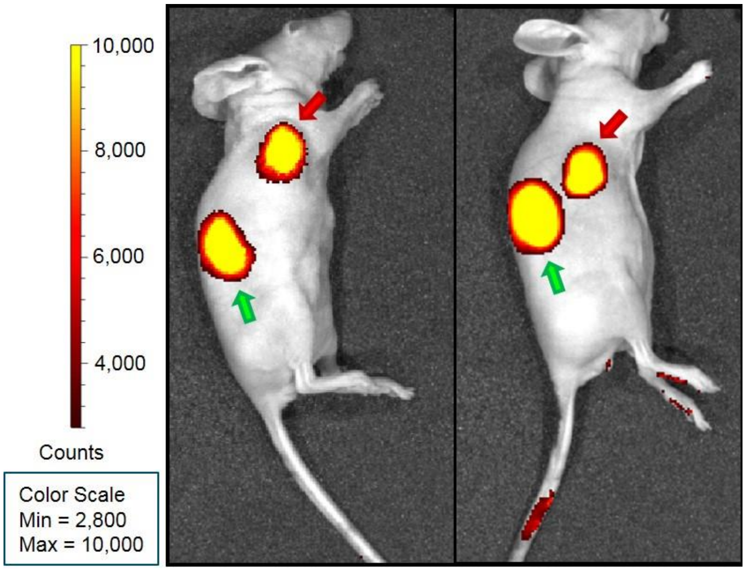

Supplemental Figure 3: NIR fluorescent images of the additional two BALB/c nude mice bearing subcutaneous CHL GLP-1R tumors. Tumors are indicated with red arrows and kidneys with green arrows.
